# RETRACTED: Xiang et al. Schisandrin B Induces Apoptosis and Cell Cycle Arrest of Gallbladder Cancer Cells. *Molecules* 2014, *19*, 13235–13250

**DOI:** 10.3390/molecules30244734

**Published:** 2025-12-11

**Authors:** Shan-Shan Xiang, Xu-An Wang, Huai-Feng Li, Yi-Jun Shu, Run-Fa Bao, Fei Zhang, Yang Cao, Yuan-Yuan Ye, Hao Weng, Wen-Guang Wu, Jia-Sheng Mu, Xiang-Song Wu, Mao-Lan Li, Yun-Ping Hu, Lin Jiang, Zhu-Jun Tan, Wei Lu, Feng Liu, Ying-Bin Liu

**Affiliations:** 1 Department of General Surgery, Xinhua Hospital, Affiliated to Shanghai Jiao Tong University School of Medicine, No. 1665 Kongjiang Road, Shanghai 200092, China; xiangshanshan@sjtu.edu.cn (S.-S.X.); surgwxa@gmail.com (X.-A.W.); l.hf.ok@163.com (H.-F.L.); shuyijun19881125@163.com (Y.-J.S.); baorunfa163@163.com (R.-F.B.); fei_zhang_1_2_6@126.com (F.Z.); cycaoyang@163.com (Y.C.); yeyuanyuan19891028@126.com (Y.-Y.Y.); wenghao1987@126.com (H.W.); wuwenguang08@126.com (W.-G.W.); good_surgeon@hotmail.com (J.-S.M.); wuxiangsong_1983@sina.com (X.-S.W.); limaolan6@163.com (M.-L.L.); kevintony@126.com (Y.-P.H.); jianglin1987_jl@126.com (L.J.); tanzj1981@126.com (Z.-J.T.); wellu@163.com (W.L.); 2 Laboratory of General Surgery, Xinhua Hospital, Affiliated to Shanghai Jiao Tong University School of Medicine, No. 1665 Kongjiang Road, Shanghai 200092, China; 3 Institute of Biliary Tract Disease, Shanghai Jiao Tong University School of Medicine, No. 227 South Chongqing Road, Shanghai 200025, China; 4 The First Affiliated Hospital Nanchang University Emergency Unit, No. 17 Yongwai Road, Nanchang 330006, China

The journal retracts the article, titled “Schisandrin B induces apoptosis and cell cycle arrest of gallbladder cancer cells” [1], cited above.

Following publication, the authors contacted the Editorial Office to raise concerns relating to the integrity of an image presented in this publication [1].

Adhering to our standard procedure, the Editorial Office and Editorial Board conducted an investigation that confirmed indications of inappropriate editing and partial duplication of Western blots within Figure 5 [1]. While the authors collaborated with the Editorial Office during the investigation, they were unable to satisfactorily explain the overlapping visual features, and the Editorial Board could not confirm the validity of the images from the material provided. As a result, the Editorial Office, Editorial Board, and authors have decided to retract this article as per MDPI’s retraction policy (https://www.mdpi.com/ethics#_bookmark30).

This retraction was approved by the Editor-in-Chief of the journal *Molecules*.

The Authors agreed to this retraction and indicated their intention to re-publish the manuscript after revisions.
